# Long Non-Coding RNA (lncRNA) in Oral Squamous Cell Carcinoma: Biological Function and Clinical Application

**DOI:** 10.3390/cancers13235944

**Published:** 2021-11-26

**Authors:** Jianfei Tang, Xiaodan Fang, Juan Chen, Haixia Zhang, Zhangui Tang

**Affiliations:** 1 Hunan Key Laboratory of Oral Health Research, Central South University, Changsha 410008, China; jianfeit614@csu.edu.cn (J.T.); 9320032fangxd@csu.edu.cn (X.F.); chenjuan1993@csu.edu.cn (J.C.); 2 Hunan 3D Printing Engineering Research Center of Oral Care, Central South University, Changsha 410008, China; 3 Hunan Clinical Research Center of Oral Major Diseases and Oral Health, Central South University, Changsha 410008, China; 4 Xiangya Stomatological Hospital, Central South University, Changsha 410008, China; 5 Xiangya School of Stomatology, Central South University, Changsha 410008, China; 6 The Oncology Department of Xiangya Second Hospital, Central South University, Changsha 410008, China

**Keywords:** OSCC, long non-coding RNAs, cancer, tumor microenvironment, biomarkers

## Abstract

**Simple Summary:**

Increasing evidence has revealed the regulatory roles of long non-coding RNAs (lncRNAs) in the initiation and progress of oral squamous cell carcinoma (OSCC). As some novel lncRNA-targeted techniques combined with immune checkpoint therapies have emerged, they provide a new strategy for OSCC treatment. This review summarizes current knowledge regarding the involvement of lncRNAs in OSCC along with their possible use as diagnostic and prognostic biomarker and therapeutic targets.

**Abstract:**

Oral squamous cell carcinoma (OSCC) is a type of malignancy with high mortality, leading to poor prognosis worldwide. However, the molecular mechanisms underlying OSCC carcinogenesis have not been fully understood. Recently, the discovery and characterization of long non-coding RNAs (lncRNAs) have revealed their regulatory importance in OSCC. Abnormal expression of lncRNAs has been broadly implicated in the initiation and progress of tumors. In this review, we summarize the functions and molecular mechanisms regarding these lncRNAs in OSCC. In addition, we highlight the crosstalk between lncRNA and tumor microenvironment (TME), and discuss the potential applications of lncRNAs as diagnostic and prognostic tools and therapeutic targets in OSCC. Notably, we also discuss lncRNA-targeted therapeutic techniques including CRISPR-Cas9 as well as immune checkpoint therapies to target lncRNA and the PD-1/PD-L1 axis. Therefore, this review presents the future perspectives of lncRNAs in OSCC therapy, but more research is needed to allow the applications of these findings to the clinic.

## 1. Introduction

OSCC originates in the surface of oral mucosal epithelium. It is one of the most commonly diagnosed lethal malignancies with poor prognosis worldwide. According to the Global Cancer Statistics 2020, about 53,260 new cases and 10,750 deaths of oral cancer were calculated in the USA, accounting for approximately 4% of all male cancer cases [1], and the most recent data indicate that the overall cases will rise to 510,948 in 2035 [2]. To date, surgical treatment combined with radiation and chemotherapy remains the primary approach for management of OSCC [3]. Advancements in treatment strategies such as drug development and computer-assisted surgery were expected to lead to an improved survival in OSCC patients. However, OSCC remains an incurable malignance and treatment-associated outcomes are still unchanged. In addition, most patients with OSCC are diagnosed at advanced stages and no early screening strategy has proven to be effective. Therefore, to further improve efficiency of diagnosis and acquire a better prognosis, comprehensive investigation aiming to elaborate molecular mechanisms of OSCC and to discover novel diagnostic tools and precision therapeutic approaches are still urgently needed.

Long non-coding RNAs (lncRNAs) is a novel class of RNAs with a length longer than 200 nucleotides. These RNAs cannot encode proteins or peptides [4,5]. In 1989, a study found that H19 was the first lncRNA that is highly expressed in mouse embryo and neonatal liver. The mouse H19 gene shared high homology (77%) with human H19 gene, though the specific function of the gene remains unclear [6]. Therefore, lncRNAs were mainly considered as “junk transcripts” that do not generate any function for many years. The functional research of lncRNAs has endured a long history. Luisa et al. confirmed that the H19 gene acts as a trans regulator gene of the imprinted gene network (IGN) such as Igf2 and other imprinted genes in the mouse embryo [7]. Up to now, lncRNA microarray and whole-genome transcriptome have identified more than 50,000 lncRNAs, some of which have been functionally characterized and revealed tissue-specific expression patterns [8]. With regard to their biological role in multiple human cancers including OSCC, many studies have shown that lncRNAs perform modulatory functions that influence cell biological behaviors, immune response, and transformed phenotype in cells. For example, lncRNA HOTAIR was reported as an oncogene that was pervasively overexpressed in most solid cancers including oral cancer and acts to promote OSCC invasion and metastasis [9]. THRIL is an immunoregulatory lncRNA that was shown to regulate the expression of pro-inflammatory cytokine TNF-α in Kawasaki disease and other immune-related inflammatory diseases through interacting with hnRNPL forming an RNA—protein complex to bind TNF-α promoter [10]. A study reported that the lncRNA loc100506114 contributes to the functional transformation of fibroblasts to cancer-associated fibroblasts (CAFs) in OSCC [11]. Moreover, lncRNAs are richly distributed in body fluids including blood, urine, saliva even exosomes, therefore they could be regarded as a type of non-invasive biomarker [12]. LncRNAs are functional transcripts that will help to identify various cancer characteristics and hallmarks, and further become an attractive potential therapeutic target.

In general, precise diagnosis and individual treatment is the ultimate direction in which we would like to explore, and lncRNAs have significant potential clinical values for diagnosis and treatment of OSCC. Therefore, this review summarizes the functions and molecular mechanisms of lncRNA in OSCC. In addition, we highlight the crosstalk between lncRNA and the tumor microenvironment, and discuss the potential applications that lncRNAs can serve including diagnostic and prognostic tools as well as therapeutic targets in OSCC.

## 2. Roles of LncRNA in OSCC

**Figure 1:**
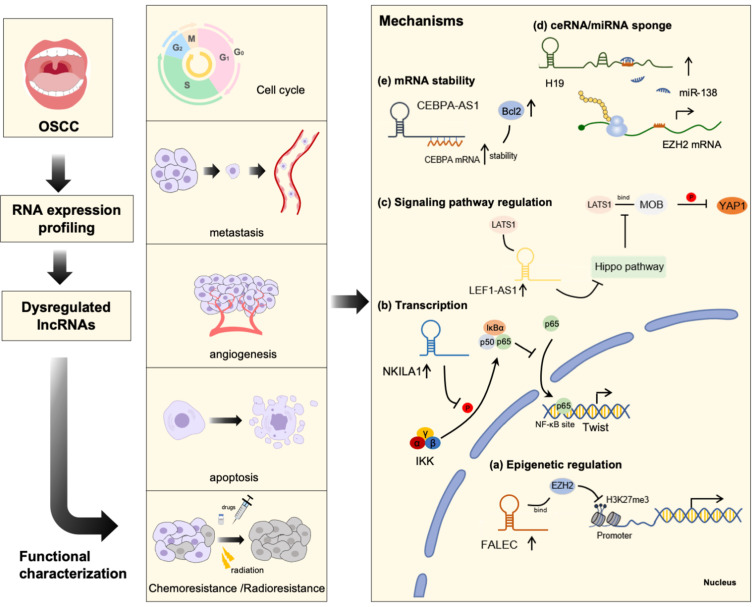
Long non-coding RNA in OSCC beginning from identification to characterization of their functions and relevant mechanisms. The first step is to acquire samples of primary tumor and normal tissues from OSCC patients, and then RNA sequencing is applied to screen dysregulated lncRNAs. Furthermore, a series oncogenic and tumor-suppressor functions in tumor biological behaviors such as cell cycle, metastasis, angiogenesis, apoptosis, and drug resistance were identified in vitro and in vivo. Mechanistically, lncRNAs regulate OSCC biological behaviors via the following aspects: (**a**) Epigenetic regulation. For example, FALEC can recruit polycomb complex EZH2 to specific genomic loci, where they methylate H3K27me3 to induce chromatin compaction and affect transcriptional activity. (**b**) Transcription regulation. For example, NKILA interacts with transcription factors NF-κB to block the action of transcription factors, thus repressing Twist expression. (**c**) Signaling pathway regulation. LEF1-AS1 interacts with LATS1 protein to inhibit the Hippo signaling pathway, resulting in attenuation of YAP1 phosphorylation (**d**) ceRNA mechanism/miRNA sponge. For example, H19, acting as a molecular sponge, competitively binds to miR-138, thereby upregulating the level of miR-138 target gene. (**e**) mRNA stability. For example, CEBPA-AS1 binding to CEBPA mRNA enhances its stability, leading to a gain of target transcripts expression.

In the past few decades, due to the emergence of high-throughput sequencing technologies, exponential growth in the number of lncRNAs with aberrant expression have been confirmed by RNA-Seq and lncRNA-microarray profiling in various cancers [13]. Fang et al. performed RNA-Seq to profile lncRNA expression in five pairs of OSCC tissues and adjacent-normal tissues; 2915 lncRNAs were significantly differentially expressed, and of these lncRNAs, 11 were associated with OSCC metastasis [14]. Based on The Cancer Genome Atlas (TCGA) database, RNA sequencing analysis of 523 oral cancer samples in India by Ganesan Arunkumar identified 11 dysregulated lncRNA in OSCC that are closely related to tobacco chewing/smoking history [15]. Evidence also showed that different functional studies revealed the role of lncRNAs in oncogenesis, tumor-suppression, and chemoresistance as well as governing virtually every physiological cell process (Figure 1). Abnormal lncRNAs are involved in many aspects of cancer cell processes including cell proliferation, apoptosis, invasion and metastasis [16], epithelial–mesenchymal transition (EMT), and drug resistance [17]. Moreover, lncRNAs even affect the outcome of patients such as lymph node metastasis, distal metastasis, and postoperative recurrence [18]. Thus, in this part, we summarize the current dysregulated lncRNAs in OSCC and elaborate on the functions of these lncRNAs in OSCC (Table 1).

### 2.1. Oncogenic Function of LncRNAs in OSCC

According to previous studies, a majority of dysregulated lncRNAs exhibit a trend of upregulated expression and function as an oncogene in promoting malignant biological behaviors in OSCC including cell proliferation, migration, metastasis, and angiogenesis. However, lncRNAs inhibited apoptosis and the process of the cell cycle. For example, many lncRNAs such as H19 [47], OIP5-AS1 [85], DNM3OS [14], AFAP1-AS1 [21], ADAMTS9-AS2 [20], LINC00668 [67], and BANCR [23] were confirmed to be overexpressed in OSCC cells and promoted tumor development by enhancing the proliferation and migration in vitro and in vivo. Beyond this, lncRNAs were also involved in the regulation of the cell cycle and inhibiting apoptosis in OSCC. For example, when the lncRNA LEF1-AS1 was silenced, it caused the arrest of the G0/G1 cell cycle and suppressed cell proliferation and growth in vitro via inactivation of the Hippo signaling pathway [59]. Furthermore, some certain lncRNAs were verified to promote OSCC invasion, metastasis, and angiogenesis [62,103]. For example, MALAT-1 is closely related to the growth and metastasis of OSCC cells and through the regulation of target small proline-rich protein (SPRR) in order to promote distant metastasis [77]. LINC00319 is downstream of Chemokine ligand 18 (CCL18), and overexpression of LINC00319 regulated the expression of VEGFA and MMP-9 to promote the angiogenic ability of OSCC cells [62]. Similarly, knockdown of FOXCUT led to the downregulation of angiogenesis factor VEGFA in Tca8113 and SCC9 cells, which indicated the potential function for FOXCUT in angiogenesis of OSCC [43].

### 2.2. Tumor-Suppressor Function of LncRNAs in OSCC

LncRNA can be used not only as an oncogene to promote the occurrence and development of tumors, but also as a suppressing factor to inhibit the growth and metastasis [104]. According to previous studies, lncRNA NKILA has been reported to be a tumor suppressor, which has been negatively correlated with metastasis and prognosis in breast cancer [105]. Consistent with the results in breast cancer, Huang et al. confirmed that NKILA expression levels in tongue squamous cell carcinoma (TSCC) was expressed significantly less. High expression of NKILA represses EMT and migration in Tscca and CAL27 cells via activation of the NF-κB/Twist signaling pathway to regulate the biological process of TSCC [84]. Growth-arrest-specific transcript 5 (GAS5) is another representative lncRNA that has been widely reported as a tumor suppressor in many cancers; Zeng et al. also confirmed that GAS5 functions as a tumor suppressor in OSCC via the miR-21/PTEN axis to inhibit tumor cell proliferation, migration, invasion, and EMT [46]. Moreover, maternally expressed gene 3 (MEG3) is an acknowledged tumor suppressor that has widely been investigated in cancers including OSCC. The overexpression of MEG3 decreases proliferation and migration of SCC15 while inducing CAL27 apoptosis. Mechanistically, MEG3 could exert the tumor-suppressor function not only by blocking the WNT/β-catenin signaling pathway, but also by acting as a miRNA sponge of miR-548d-3p to modulate the JAK–STAT signaling pathway [80,81]. Furthermore, other lncRNAs such as FALEC was also reported as a tumor-suppressor with low expression in OSCC, and the overexpression of FALEC significantly repressed OSCC cell proliferation and migration both in vitro and in vivo, and this predicts a good prognosis in OSCC patients [40]. Compared to the oncogenic functions of lncRNAs, the studies of lncRNA action as a tumor suppressor in order to exert inhibitive functions in OSCC are limited. Therefore, relevant research in OSCC requires comprehensive investigation.

### 2.3. OSCC LncRNAs Regulate Chemoresistance and Radiosensitivity

Adjuvant radiation or chemotherapy plus radiation has been the primary approach for the treatment of OSCC patients depending on the disease stage [3]. Cisplatin (CDDP) is a platinum-based drug that is commonly used as an efficient adjuvant treatment for OSCC patients. However, cisplatin-resistance is a headache in chemotherapy, resulting in tumor relapse and poor prognosis [53]. Emerging evidence showed that lncRNAs may function as vital regulators of chemoresistance in OSCC [17,106]. For instance, the upregulation of HOXA11-AS significantly increased resistance to cisplatin and tumor cell growth, and the knockdown of HOMA11-AS markedly enhanced CDDP-mediated tumor inhibition in vivo [53]. Likewise, Lin et al. reported the increased expression of CILA1 in cisplatin-resistant OSCC cells lines, and the silence of CILA1 significantly inhibited the migration, invasion, and EMT, while increasing the sensitivity to chemotherapy of these cells [36]. Furthermore, another study shown by Fang et al. indicated that UCA1 increased the proliferation of OSCC cells and induced cisplatin resistance by modulating the expression of the miR-184 target gene SF1 [101]. Notably, Zhang et al. confirmed that overexpression of lncRNA ANRIL can be induced by paracrine action of CAF-derived Midkine, thereby enhancing the proliferation and resistance to cisplatin of tumor cells [22]. Interestingly, Wang et al. confirmed that silencing HOTAIR significantly enhanced sensitivity to CDDP while inhibiting tumor cell autophagy [51]. Moreover, other lncRNAs such as KCNQ1OT1 [56,57]. LHFPL3-AS1 [60] and PVT-1 [90] were also reported to be involved in promoting cisplatin-resistance in OSCC cells.

In addition to chemoresistance, radiosensitivity is another tremendous challenge among the comprehensive therapies for OSCC. Accumulating evidence also revealed the crucial roles of lncRNAs in radiotherapy. For example, Gou et al. observed that lncRNA BLACAT1 was associated with low radiosensitivity and poor outcomes of HNSCC patients, and they further confirmed that the knockdown of BLACAT1 in SCC25 cells markedly improved the radiosensitivity by regulating PSEN1 [24]. Likewise, another example is LINC00473, which is highly expressed in OSCC cells. LINC00473 knockdown in these cells significantly enhanced the sensitivity of radiotherapy by modulating the Wnt/β-catenin signaling pathway [65].

## 3. Molecular Mechanism of LncRNAs in OSCC

In the past few years, research has revealed that lncRNAs could potentially be involved in the tumorigenesis of cancer, and identifying the molecular mechanisms of lncRNAs within OSCC is still a challenge. In general, a myriad of studies have confirmed that lncRNAs exert regulatory functions, mainly via the following mechanisms: (1) epigenetic regulation; (2) transcriptional regulation; and (3) post-transcriptional regulation [107,108]. Mechanistic studies published to date indicate that the dysregulated lncRNA in OSCC may exert their biological functions through these molecular mechanisms (Figure 1).

### 3.1. Epigenetic Regulation

Epigenetic regulation is a complex process, which is mediated by DNA and histone modifications, and are crucial for transcription machinery during gene expression [107,109]. Recent studies have clearly suggested that epigenetic changes can contribute to the development of several human malignancies and that lncRNAs play a vital role in this context. A battery of lncRNAs have been reported to mediate chromatin remodeling and DNA modification by acting as a molecular scaffold, thereby regulating the expression of genes before finally affecting cancer development [110].

A typical example is the association with polycomb repressive complexes 2 (PRC2), which is composed of EED, SUZ12, and EZH2 [111]. It can catalyze H3K27 trimethylation, causing chromatin compaction and thereby affecting the transcriptional state. To date, lncRNA HOTAIR has been shown to promote migration, invasion, and poor survival in OSCC. HOTAIR could act as a molecular scaffold to recruit EZH2 and H3K27me3, thereby repressing E-cadherin expression [9]. Another study verified that lncRNA FALEC could also recruit PRC2 component EZH2, causing H3K27me3 trimethylation and ECM1 silencing, leading to the inhibition of proliferation and migration in OSCC cells [40]. Furthermore, dysregulation of chromatin modification can modulate lncRNA transcription, cancer initiation, and malignant progression. One recent study indicated that lncRNA PLAC2 is transcriptionally activated by CBP-mediated H3K27 acetylation at the promoter region and promoted OSCC progression via activation of the Wnt/β-catenin signaling pathway [89]. There are still other patterns of epigenetic modifications mediated by lncRNAs in OSCC urging further exploration.

### 3.2. Transcriptional Regulation

In addition to the above features of lncRNA epigenetic regulation, lncRNAs could also regulate gene expression in a transcription-dependent manner, which leads to cancer progression [111]. Recent studies indicate that lncRNAs can regulate transcription to recruit TFs or by directly binding to the gene promoters, thereby regulating different malignant biologic behaviors of tumors. On one hand, lncRNAs interact with key cancer associated TFs to regulate the activity of transcription. One study reported that tumor suppressor lncRNA NKILA hindered OSCC migration and invasion by interacting with NF-κB and ultimately lowering Twist and E-cadherin [84]. On the other hand, by interacting with DNA-binding proteins including CTCF, lncRNAs can facilitate their binding and expression at a targeted gene region. The DNA-binding protein CTCF can mediate chromatin interaction and DNA looping [112]. This type of mechanism can be observed in LINC00941, which drives CTCF recruitments to promote CAPRIN2 expression, thereby accelerating cell proliferation and colony formation in OSCC [69]. However, in recent studies, scholars have reported that some enhancers can also produce enhancer RNA (eRNA) through transcription, which can modulate genes that are far away from a specific direction, but the specific relationship between enhancer genomic loci and gene expression regulation remains unclear, especially in OSCC; related studies need to be further explored [107].

### 3.3. Post-Transcriptional Regulation

Emerging reports have indicated that lncRNA functions as a post-transcriptional regulator in the gene expression process. The mechanisms of lncRNA in post-transcriptional regulation mainly include the alteration of mRNA splicing, miRNA sponge, mRNA stability, protein translation, and RNA editing, even in the regulation of signaling pathways [4]. lncRNA regulated phosphorylation of critical signaling molecules have been reported to contribute to cancer progression. For instance, lncRNA LEF1-AS1 could interact with the LATS1 protein, leading to attenuated YAP1 phosphorylation that ultimately promotes cell proliferation and migration in OSCC. Further studies have demonstrated that depletion of lncRNA LEF1-AS1 results in upregulation of cytoplasmic YAP1 expression and downregulation of the nuclear YAP1 level, suggesting the involvement of lncRNA LEF1-AS1 in the regulation of phosphorylation of the crucial players of the Hippo signaling pathway [59]. On the other hand, lncRNAs regulate mRNA stability via post-translational modifications that support cancer development. A recent study showed that LINC00284 facilitates the mRNA stability of KAZN by binding with RBP FUS and promoting cell proliferation and migration in OSCC [61]. Notably, lncRNA FOXC1 associates and forms a lncRNA–mRNA duplex with FOXCUT and thereby increases its stability to promote OSCC cell migration [43]. Likewise, lncRNA CEBPA-AS1 could bind with CEBPA mRNA, thus leading to OSCC cell proliferation, invasion, and migration [35].

In addition, lncRNA was shown to function as a competing endogenous RNA (ceRNA) and regulate gene expression at the posttranscriptional level, consequently influencing cancer progression [113]. In detail, lncRNA could use its miRNA response elements (MREs) in mRNA binding site as a natural decoy for miRNA and inhibits the ex-pression of miRNA on the target gene (mRNA) [113,114,115]. Recently accumulating evidence has confirmed the role of lncRNA in regulating the pathogenesis of OSCC via miRNA sponge. For example, lncRNA RC3H2 facilitates OSCC tumor growth and metastasis by acting as a ceRNA for miRNA-101-3p. Mechanistically, lncRNA RC3H2 can compete with miRNA-101-3p for binding with the target EZH2 to promote OSCC malignant behavior [92]. Another ceRNA, lncRNA H19, acts by competitively sponging miR-138 and upregulating vimentin and N-cadherin expression, leading to EMT in OSCC [47]. Additionally, lncRNA KCNQ1OT1 promotes cisplatin resistance of OSCC by functioning as a molecular sponge for miR-211-5p to activate Ezrin/Fak/Src signaling [56]. Other lncRNAs such as LTSCCAT [76], OIP5-AS1 [85], DNM3OS [14], SNHG16 [95], HOTTIP [52], HCP5 [50], JPX [55], and AFAP1-AS1 [21] were also reportedly involved in promoting the progression of OSCC via the ceRNA mechanism. In summary, lncRNAs could act as ceRNAs to sponge various miRNAs to participate in post-transcription, thus promoting OSCC malignant development.

Collectively, it is important to note that individual lncRNAs could exert their functions through different modes of post-transcriptional regulation simultaneously. The regulation of other posttranslational modifications including pre-mRNA alternative splicing by lncRNAs has not been substantiated in OSCC. Future work will be aimed at elucidating the mechanism of action of lncRNAs that use other posttranslational pathways in contributing to OSCC development.

## 4. The Crosstalk between LncRNA and Tumor Microenvironment in OSCC

**Figure 2:**
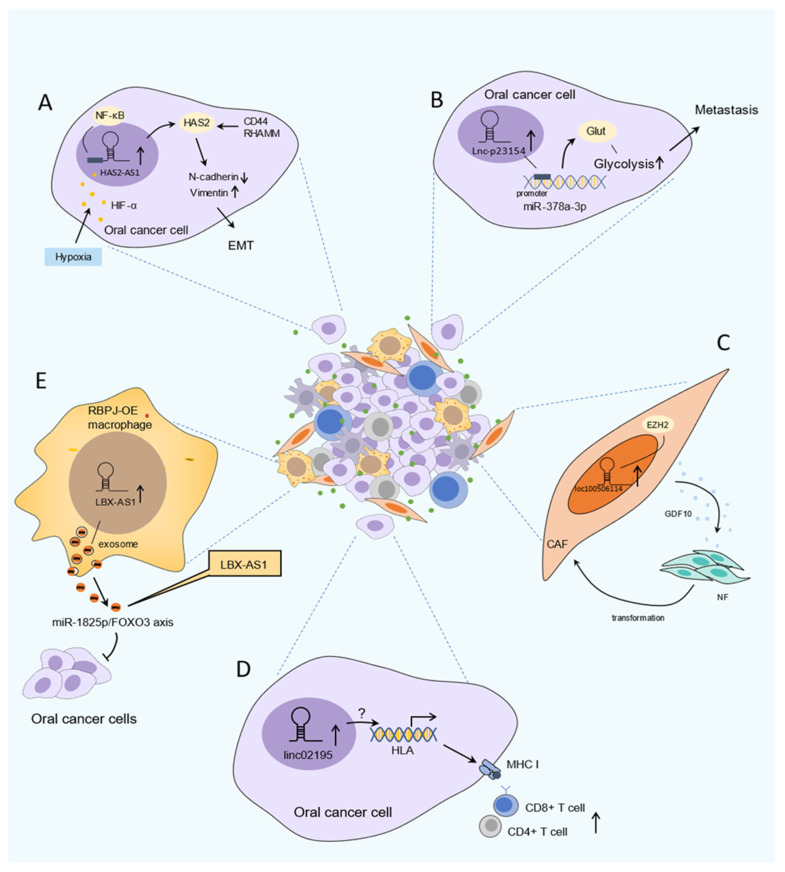
Roles of lncRNA in OSCC tumor microenvironment. (**A**) Hypoxic TME. HAS2-AS1 activates NF-κB in response to HIF-1α induced by microenvironment hypoxia, leading to HAS2 accumulation in CD44, RHAMM depend-way, which promotes EMT and invasion. (**B**) Metabolic reprogramming. lncRNA-p23154 promotes GLUT1 expression to enhance glycolysis via binding with the promoter region of miR-378a-3p, leading to increased metastatic potential. (**C**) CAF transformation. loc100506114 promotes functional transformation of NFs to the phenotype of CAFs by forming a feedback loop with EZH2 to activate CAFs secrete GDF10. (**D**) Immune regulation. Linc02195 regulates MHC I protein to affect immunosurveillance by being closely associated with high expression of HLA I gene, and it showed a positive correlation between increasing number of infiltrating CD8+T and CD4+T cells. (**E**) Extracellular vesicles. Exosomal-LBX1-AS1 from RBPJ overexpressed macrophages inhibited the proliferation and invasion of OSCC cells by the miR-182-5p/FOXO3 pathway.

TME is mainly composed of parenchyma cells, immune cells, peripheral extracellular matrix (ECM) as well as some signal molecules [116]. TME of OSCC is characterized by hypoxia, chronic inflammation, and immunosuppression. This surrounding environment is regarded as an intricate physical and biochemical system, which is involved in tumor onset, progression, metastasis, and influences the prognosis of treatment. In particular, recent emerging studies have indicated that abnormal expression of certain lncRNAs is strongly associated with hypoxia, metabolism, immune cells, and CAFs; therefore, these lncRNAs have a crucial role in TME (Figure 2).

### 4.1. LncRNA and Hypoxic, Metabolic TME in OSCC

Hypoxia is a common and important feature in the TME and is tightly linked to cancer development and aggressive phenotypes [117]. Under hypoxic TME, the vital regulator Hypoxia-induced factor-1 alpha (HIF-1α) mediates tumor growth, invasiveness, and metastasis, contributing to aggressive phenotypes in various cancers including OSCC [82]. Despite hypoxic response signaling having been extensively explored, the involvement of lncRNAs in the hypoxic response has become a new focus of cancer research. It is well established that 56 hypoxia-associated lncRNAs (HALs) have led to cancer progression [117]. HALs have been analyzed in OSCC research and found to be associated with worse outcome and clinicopathological characteristics [82]. Zhu et al. found that lncHAS2-AS1 was substantially increased in OSCC. In response to hypoxic conditions, HIF-1α drove the expression of lncHAS2-AS1, which caused HAS2 accumulation. Moreover, the study also demonstrated that lncHAS2-AS1 promoted the EMT and invasion potential in OSCC [49]. Notably, expression of HALs also regulate HIF-1α activity. Shih et al. showed that the expression level of lncHIFCAR is significantly elevated in OSCC, which is associated with tumor grade as well as poor overall survival and recurrence-free survival. In response to hypoxic conditions, lncHIFCAR directly binds with HIF-1α and further facilitates HIF-1α target expression, leading to increased invasion, metastasis, metabolic reprogramming, and sphere-forming ability in vitro and in vivo [82].

Notably, recent studies have revealed that some lncRNAs were involved in reprogramming metabolism, especially modulating glycolysis in OSCC cells, resulting in the progression of malignant behaviors such as proliferation and metastasis [39,86,118]. A representative example is lncRNA-p23154. It enhances metastasis in OSCC through binding with the promoter region of miR-378a-3p in the nucleus in order to promote glucose transporter 1(GLUT1) expression, leading to enhanced glycolysis [86]. Moreover, ELF3-AS1 is another typical lncRNA, which is upregulated in OSCC; the silencing or forced expression of ELF3-AS1 in OSCC cells resulted in the same trend of GLUT1 expression; ELF3-AS1 and GLUT1 overexpression leads to a significantly increased proliferation rate of OSCC cells and glucose uptake, which means that upregulation of ELF3-AS1 promotes the proliferation of OSCC cells, and may positively regulate GLUT1 to affect glucose metabolism [39]. Likewise, Yang et al. reported that H-19 was associated with glycolysis in oral CAFs via the interaction of H19-derived miR-675-5p binding to PFKFB3, resulting in the proliferation and migration of OSCC cells [118].

### 4.2. LncRNA and Cancer-Associated Fibroblasts

CAFs are important and abundant components within TME, which interact closely with tumor cells by a paracrine mode of action, thus contributing to tumor initiation and malignant progression [119,120]. It has been reported that lncRNAs are involved in and sustain this interaction, and this effect on the TME has aroused extensive attention.

Under the action of lncRNA, normal fibroblasts (NFs) were activated and acquired CAF phenotype, which in turn promotes many aggressive features in cancer including OSCC. Ding et al. revealed that upregulated FLJ22447 maintains the stromal phenotype of CAF via IL-33, thus promoting tumor proliferation [42]. Moreover, other examples of lncRNAs playing a key role in CAFs within OSCC are TIRY and loc100506114, and have been described as being elevated in CAFs when compared to the adjacent NFs. Two studies demonstrated that TIRY and loc100506114 participated in the functional transformation of human NFs to the phenotype of CAFs, which supports tumor cell growth, invasion, and metastasis [11,97].

Interestingly, CAFs have been reported to induce upregulation of lncRNA in tumor cells. In this regard, Ding et al. showed that OSCC cells expressed FLJ22447 in an exosomal manner to activate adjacent NFs, thereby facilitating elevated FLJ22447 to obtain the phenotype of CAFs, which form a positive feedback loop to promote OSCC development, which are associated with short survival and poor prognosis in OSCC patients [42]. In another example, Midkine secreted by CAFs promoted the upregulation of lncRNA ANRIL in OSCCs. Such lncRNA was reported to enhance cisplatin-based chemoresistance [22]. Additionally, Yang et al. reported lncRNA H19 as potential epithelial–mesenchymal common targets (EMCTs) and is involved in regulating glycolysis, proliferation, and migration in oral CAFs via the miR-675-5p/PFKFB3 pathway [118]. Altogether, lncRNA has been shown to play a role in the crosstalk between CAFs and OSCC cells and may be regarded as a possible therapeutic target or predictive biomarker.

### 4.3. LncRNA and Cancer-Associated Immune Cells in OSCC

Infiltrated immune cells such as T cells, tumor-associated macrophages (TAMs), dendritic cells (DCs), and natural killer cells (NKs) are also key components of TME [3]. The reciprocal crosstalk between cancer cells and immune cells shapes the pro-tumorigenic microenvironment in a way that renders it to escape immune surveillance and suited for immune tolerance [121]. LncRNAs are reported to participate in various processes of immune response within TME to promote tumor progression [122]. In OSCC patients, Feng et al. found that lncRNA SLC16A1-AS1 was positively correlated with resting NK cells, M1 macrophages, activated mast cells, and activated memory CD4 T cells by bioinformatics analysis, but negatively correlated with plasma cells, T follicular cells, resting mast cells, and Tregs [93]. Likewise, Li et al. reported that LINC02195 was an immune-related lncRNA that was upregulated in OSCC cells. A positive correlation can be seen between increasing number of infiltrating CD8+T, CD4+T cells, and LINC02195 by bioinformation analysis. In addition, LINC02195 acts as a regulator, which was closely associated with high expression of the HLA I gene, thereby regulating the MHC I protein to show the potential function in affecting immunosurveillance [75]. Moreover, the silence of lncRNA TUG1 substantially enhanced NK cells, killing sensitivity in OSCC cells [123]. Notably, lncRNA LBX1-AS1 was significantly upregulated in the exosomes, which are derived from RBPJ overexpressed-macrophages in OSCC, and lncRNA LBX-AS1 inhibits tumor growth and invasion. Furthermore, this effect can be attenuated by lncRNA LBX1-AS1 knockdown [58]. In addition, lncRNAs can impact the function and cytotoxicity of T cells via regulation of the expression of molecules on the surface of the tumor cells and directly inducing cell death or enhancing T cell exhaustion in the TME [124]. For example, LNC-SOX5, which is associated with carcinogenesis of tongue carcinoma, has also been reported to function on the regulating cytotoxicity of CD8+ T cells in colorectal cancer [54,125], while at present, the function of LNC-SOX5 in regulating the CD8+ T cell cytotoxicity effect in OSCC remains unclear. Furthermore, Liu et al. also reported that lncRNA FOXD2-AS1 was involved in regulating the proliferation and functions of antigen-presenting cells (APCs) to inhibit the adaptive immunity in OSCC [44]. The above evidence indicates the significance of lncRNAs in immunotherapy and it could be the potential immunotherapy target. However, only a few lncRNAs have been reported in the crosstalk with immune cells in OSCC currently, and comprehensive study remains deficient.

### 4.4. Extracellular Vesicles: Exosome-Associated LncRNAs in TME

Exosomes refer specifically to a kind of extracellular vesicle (EV) that is secreted by most eukaryotic cells [126]. Currently, due to their unique functions in mediating intercellular communication and activating signaling as extracellular messengers, exosomes as effective signaling molecules have been broadly investigated in cancers [127,128]. The diverse cargo in which exosomes carry such as lncRNA are released from exosomes and will dynamically change depending on the cell type. These lncRNAs are involved in regulating tumor metastasis, angiogenesis, immunosuppression, drug resistance, and metabolism [129,130], thereby enhancing the interaction between cancer cells and surrounding cells [131]. For instance, in the studies of OSCC, FLJ22447 and LBX1-AS1 were representative exosome-associated lncRNAs that were derived from CAFs and macrophages, respectively. Both of these are strongly associated with OSCC progression, recurrence, and poor prognosis [42,58]. Analysis of FLJ22447 derived from CAFs revealed it as a medium in which to interact with surrounding stromal fibroblasts to exert the modulatory function in activating CAFs via IL-33, thus inducing the proliferation of OSCC cells [42]. In contrast, combined analysis of LBX1-AS1, which is secreted from RBPJ overexpressed macrophages in OSCC patients, the overexpression of exosome-associated lncRNA LBX1-AS1 upregulated tumor suppressor gene FOXO3 to inhibit the proliferation and invasion of OSCC cells [58]. These studies suggested that exosomes, especially exosome-associated lncRNA, probably exert modulation functions in OSCC development and could be a potential biomarker for OSCC diagnosis and therapy. However, exosome-associated lncRNA has been a novel topic in recent years. Some lncRNAs such as LINC01133 have been reported to act as a tumor repressive gene in OSCC cells [71], while LINC01133, derived from exosomes only, have been reported in pancreatic tumors and bladder urothelial carcinoma [132,133].

Current research has already revealed the roles of exosome-associated lncRNAs from different cells in OSCC patients. Therefore, exosome-associated lncRNAs might be potential biomarkers for the diagnosis and therapy of OSCC. Furthermore, compared with conventional targeting vectors, exosomes have lower systemic toxicity and higher stability, and exhibit nonimmunogenic properties [134]. For this purpose, the introduction of engineered lncRNAs into OSCC cells or tissues might represent a new and efficient approach for future cancer therapy.

## 5. Translational Potential of LncRNAs in OSCC

**Figure 3:**
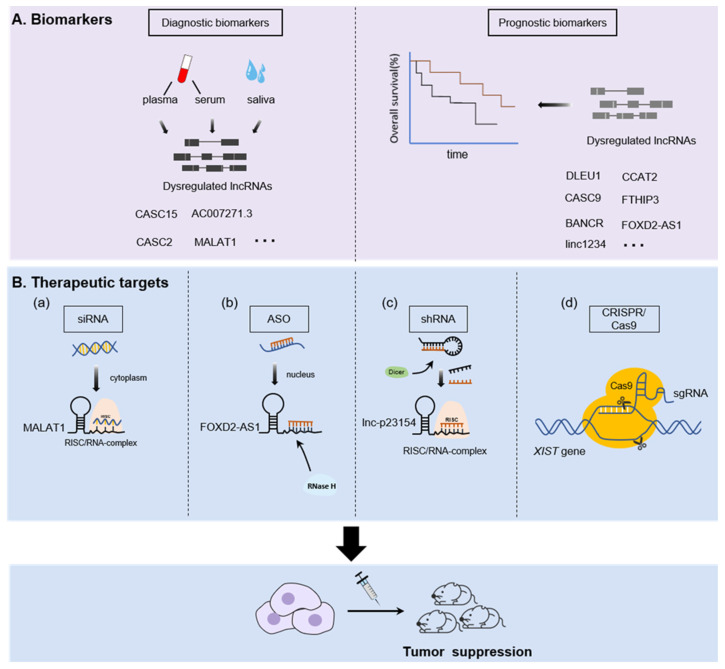
Potential clinical application of lncRNAs in OSCC. (**A**). lncRNAs as diagnostic and prognostic biomarkers; (**B**) lncRNAs as therapeutic targets based on four main lncRNA-based techniques. (**a**) MALAT1 silenced by siRNA in cytoplasm of Tscca and Tca8113 cells cause the inhibition of invasion and migration. (**b**) ASO. ASO targets FOXD2-AS1 in the nucleus of CAL27cells, leading to inhibition of tumor growth. (**c**) shRNA. shRNA targeting lnc-p23154 and transfected into HSC-3 cells led to the inhibition of tumor metastasis. (**d**) CRISPR/Cas9 system. Guided by the sgRNA, Cas9 can specifically knock out the sequence of the XIST gene in SCC9 cells, leading to the tumor suppressor function.

The presence of numerous lncRNAs that may have roles in cancer progression and outcomes have important clinical implications. On one hand, lncRNAs are stable and widely distributed in various tissues and body fluids including blood, saliva, and urine, making them a promising noninvasive biomarker for cancer diagnosis and prognosis [135]. On the other hand, lncRNAs have highly tissue-specific expression patterns and are functionally characterized, which contribute to the hallmarks of cancer [136]. Therefore, they are potential therapeutic targets. Here, we present the translational potential of lncRNAs in OSCC (Figure 3).

### 5.1. LncRNAs as a Novel Diagnostic and Prognostic Tools

Studies have confirmed that body fluids can detect the dysregulated lncRNA from primary tumors [135]. A representative lncRNA is prostate Cancer Antigen 3(PCA3, also known as DD3), which is derived from the patient’s urine and is widely applied in the diagnosis of prostate cancer due to its high specificity and sensitivity [137,138,139]. With regard to OSCC, Jia et al. confirmed that the expression profile of plasma lncRNAs in OSCC patients are changed by microarray analysis [140], which suggested the potential diagnostic value of circulating lncRNAs in OSCC. For instance, the level of CASC2 significantly decreased in the plasma of OSCC patients with local recurrence while it increased in those without recurrence; receiver operating characteristic curves (ROC) showed a significant value in detecting the expression of plasma CASC2 for OSCC diagnosis (the area under the ROC curves (AUC) = 0.8445) [28]. CASC15 is another lncRNA that was upregulated in the plasma of patients with OSCC, and this feature can also be applied to distinguish OSCC patients from oral ulcer patients [26]. In parallel, a study by Shao et al. identified lncAC007271.3, a kind of serum lncRNAs with a high expression level in OSCC patients when compared to the classic tumor markers SCCA, the ROC curves illustrated that the level of serum AC007271.3 could effectively discriminate between OSCC patients and controls (AUC = 0.873; 95% confidence interval (CI), 0.815–0.931; *p* < 0.001) with high sensitivity and specificity (77.6% and 84.5%, respectively) [141]. Moreover, a study by Tang et al. revealed that saliva in its entirety contains detectable amounts of certain lncRNAs such as HOTAIR and MALAT1 [12]. Interestingly, HOTAIR was differentially expressed in the saliva of OSCC patients with metastasis compared to those who without metastasis [12], which indicates the prospect of detecting lncRNAs in saliva to act as a rapid and noninvasive tool for OSCC diagnosis.

LncRNAs have also been indicated to be closely related to a series of clinic pathological parameters such as lymph metastasis and local recurrence in OSCC and may serve as valuable predictive biomarkers. The expression level of LINC00152 is increased in OSCC tissues and is positively correlated with cervical lymph node metastasis, higher TNM stage, and postoperative recurrence [8]. Similarly, other lncRNAs such as H19, CCAT2, and TUG1 have also been reported to have high expression levels in OSCC and are associated with the TNM stage and pathological grade [34,47,99]. In particular, one study reported that LINC-RoR was associated with cellular differentiation in OSCC, and was highly expressed in tumors with undifferentiated pathology and served as a predictor to therapeutic response [15]. Furthermore, a study by Jin et al. reported increased TIRY expression in OSCC tissues. Authors used ROC and diagnostic evaluation tests to reveal that OSCC patients with different risk of recurrence or metastasis within one year could be distinguished by TIRY expression (AUC = 0.897), which indicates the potential diagnostic value of TIRY [97].

In addition to the diagnostic potential of lncRNAs, previous studies have already confirmed that lncRNAs are associated with the survival time of OSCC patients and regarded as prognostic biomarkers. For example, MALAT1 was highly expressed in OSCC tissues and Zhou et al. verified that the lower expression of MALAT1 in OSCC patients had a better survival rate computed by Kaplan–Meier analysis [78]. Likewise, Yao et al. observed markedly upregulated BANCR expression in OSCC tissues; more importantly, a multivariate proportional hazards (COX) regression analysis revealed that in addition to lymph node metastasis, BANCR expression level was independently associated with poor overall survival (OS) and disease-free survival (DFS), which suggests BANCR was an independent prognostic factor in OSCC patients [23]. The same as BANCR, Yang et al. reported that the average OS of OSCC patients with low CASC9 expression was longer than those with high CASC9 expression by Kaplan-analysis, and COX regression analysis also revealed that the CASC9 expression level was an independent predictor of the OSCC prognosis [29]. In addition, similar reports of other lncRNAs such as LINC01234 [73,74], colon cancer–associated transcript 2 (CCAT2) [87], FOXD2-AS1 [44] and FTH1P3 [45] were also confirmed to be unregulated in OSCC tissues and associated with low OS, which indicated the poor prognostic capability.

However, tissue biopsy is still considered the gold standard for cancer diagnosis, even though it is an invasive procedure. The main advantages of lncRNAs as a biomarker for cancer diagnosis and prognosis is due to the high stability, high sensitivity, specificity, and non-invasive nature during body fluid circulation [135]. Due to the features of specificity in diseases and cell types, it is much easier to detect and make lncRNAs that are suitable for diagnosis in cancer patients. LncRNAs serve as valuable biomarkers in applications such as diagnosis and prognosis and have been shown to have tremendous potential in the future.

### 5.2. LncRNAs as Therapeutic Agents or Targets

Given the fact that the expression of lncRNAs is tissue/cell specific, and the carcinogenic roles of these lncRNAs are diverse, lncRNAs show promise as attractive targets for drug development and significant implication in clinical application for cancer treatment. Therapeutic approaches that accurately target lncRNAs may exert anti-tumor effects. Emerging advanced lncRNA-based techniques such as antisense oligonucleotides (ASOs), small interfering RNAs (siRNAs), short hairpin RNAs (shRNAs), aptamers, and CRISPR-Cas9, have revealed that targeting specific lncRNAs can effectively inhibit OSCC growth, migration, and invasion in vitro and in vivo [142].

Conventional tools such as siRNAs and shRNAs have already been extensively employed to study the mechanisms and therapy of lncRNAs. For example, intratumor delivery of MALAT1-targeted siRNA substantially inhibits tumor growth in the Tscca xenograft mouse model [78]. Knockdown of lncRNA LINC00460 by siRNA-based targeting method siLINC00460 led to inhibition of tumor metastasis in lung metastasis models in OSCC [63]. Furthermore, Wang et al. also demonstrated that tail vein injection of an shRNA specifically targeting lnc-p23154 significantly reduced tumor metastasis in the OSCC mouse model [86]. Interestingly, shRNA-mediated knockdown of lncRNA KCNQ1OT1 can increase cisplatin sensitivity as well as decrease tumor burden in OSCC xenografts, suggesting a novel potential approach for the reversion of cisplatin resistance in OSCC [56]. However, studies on siRNA technology specificity have shown off-target effects [143].

ASO techniques are the most powerful approach to target lncRNAs. For example, ASO-mediated FOXD2-AS1 silence has been shown to inhibit tumor growth in a OSCC mouse [44]. Furthermore, Li et al. observed that tumor growth and metastasis was significantly suppressed after AC104041.1 specific LNA-ASO in patient-derived xenograft (PDX) models generated from HNSCC patients [19]. However, off-target effects can be observed when targeting lncRNAs using ASO therapeutics, which can be seen in the generally low abundance of lncRNAs in vivo [142]. Despite these obstacles, protein-coding genes have been targeted using ASOs. An ASO targeting STAT3 (AZD9150) was tested for antitumor activity in patients with refractory lymphoma and lung cancer (NCT01563302) [144]. Therefore, the ASO technique of targeting lncRNAs for cancer treatment may be hopeful. Surprisingly, the CRISPR–Cas9 system has transformed the way lncRNAs are studied and has offered new opportunities for therapeutic targeting of lncRNAs in cancer research [145]. For instance, Zhang et al. confirmed that genomic deletion of lncRNA XIST using the CRISPR/Cas9 system reduced the tumor formation in tongue cancer [146]. In parallel, according to a study by Chang et al., CRISPR/Cas9 targeting MIR31HG markedly presented a reduction in oncogenicity of OSCC, which shows the potential therapeutic efficacy by targeting MIR31HG [83]. Thus, CRISPR/Cas9 may open a bright pathway for tumor therapy for gene-level tumor treatments considering its higher stability and lower off-target effect. So far, no lncRNA-targeting therapeutics have entered clinical development, and personalized and targeted therapy based on CRISPR/Cas9 is likely the future of tumor therapy.

Beyond the above mentioned statements, anti-PD-1/PD-L1 therapy has recently been approved for use in recurrent and/or metastatic OSCC patients, however, quite a number of patients are resistant to immune therapy [147]. Therapy targeting these lncRNAs may regulate the resistance within patients with OSCC to anti-PD-1/PD-L1 immunotherapy. LncRNA as a potential upstream regulator can target the PD-1/PD-L1 axis, thereby leading to marked anti-tumor activity [148]. Studies have reported that SNHG20 promotes esophageal squamous cell carcinoma growth and metastasis by activating the ATM/JAK/PD-L1 pathway [149]. GATA3-AS1 enhances the ubiquitination of PD-L1 by upregulating COPS5, thus promoting the immune escape of breast cancer cells [150]. Remarkably, interventional methods targeting UCA1 and anti-PD-1 treatment enhance therapy efficacy for bladder cancer [151]. More importantly, Ma and their team reported IFNα-induced lncMX1–215 markedly suppressed proliferation and metastasis capacity in OSCC. LncMX1-215 negatively regulated PD-L1 expression to inhibit immune escape [48]. The results can direct us toward a potential chemotherapy regime and suggests that targeting lncRNAs can be used to design more effective immune therapies by targeting the PD-1/PD-L1 axis. However, comprehensive investigation should further support the strategy.

## 6. Perspectives and Conclusions

Despite significant efforts, clinical trials testing novel treatment strategies, personalized medicine and non-invasive, specific biomarkers are the final goals to improving the survival rate of OSCC patients. It is worth noting that outstanding questions and challenges remain.

First, even though the pro-cancer and anti-cancer roles of lncRNAs are revealed in OSCC, we need to clarify different expression levels of lncRNAs in OSCC patients with diverse clinic stages, thereby providing better clues for early diagnosis and discriminating tumor progression. Second, lncRNA can easily be detected in saliva, particularly in OSCC metastatic patients. Therefore, lncRNAs derived from saliva that act as the symbolic body fluid in the oral cavity should be considered for biomarker viability. Third, lncRNAs act as regulators that are associated with TME of OSCC in diverse aspects such as hypoxic conditions, metabolic reprogramming, CAFs, immune cells, and exosomes. However, the explorations of their underlying mechanisms have only begun to scratch the surface and comprehensive studies are still deficient. Fourth, due to the poor conservation of lncRNAs in different species [136], whether or not the experimental results obtained from animal models in vivo can be extended and applied to humans needs further clinical trials to prove their efficacy. More importantly, due to some of the existing challenges in lncRNA therapeutics such as the hurdles of specificity, delivery, tolerability, and the unpredictable off-target effects of lncRNA-based techniques [143], there is still a substantial amount of information to explore. Potential solutions have been mentioned to improve these problems such as chemical modification for optimizing hepatotoxicity and off-target effects of ASOs or instead of stable viral transduction [142,143]. Some substantial progress in oligonucleotide technique is also being explored such as locked nucleic acids (LNAs) with better character of stability and low toxicity [13]. Furthermore, the novel gene-editing technology CRISPR/Cas9 system also showed tremendous potential in clinical application due to its operability and economical features [145].

Notably, lncRNAs are involved in regulating the PD-1/PD-L1 axis, which plays an important role in immunotherapy in cancer patients. However, no lncRNA-targeting therapeutics to modulate the PD-1/PD-L1 axis have entered clinical trials. Therefore, the detailed mechanisms of lncRNA modulation of the PD-1/PD-L1 axis are still poorly understood in OSCC. Further investigations will help us understand that targeting lncRNAs in combination with anti-PD-1/PD-L1 may prove to be a broadly applicable new strategy in tumor immunotherapy.

Taken together, as described in this review, we summarize the functions and potential molecular mechanisms of dysregulated lncRNAs in OSCC as well as the crosstalk with the tumor microenvironment. In particular, we emphasized the translational potential of lncRNAs in the diagnosis and treatment in the future, especially the emerging lncRNA-targeted therapeutic techniques including CRISPR-Cas9 as well as immune checkpoint therapies to target lncRNA and the PD-1/PD-L1 axis. Finally, we truly believe that we have only made the first step toward an in-depth understanding of the functions of lncRNAs in OSCC and this review presents the future perspectives of lncRNAs in OSCC therapy.

## Figures and Tables

**Table 1:** Summary of dysregulated lncRNAs and related targets in OSCC.

lncRNA	Expression	Functions	Targets	References
AC104041.1	↑	Promotes growth and metastasis	Wnt2B/β-catenin signaling	[19]
ADAMTS9-AS2	↑	Promotes proliferation, migration, EMT	miR-600/EZH2	[20]
AFAP1-AS1	↑	Promotes proliferation, migration, invasion	miR-145/HOXA1	[21]
ANRIL	↑	Promotes proliferation, drug resistance	Midkine/anti-apoptotic protein Bcl-2	[22]
BANCR	↑	Promotes proliferation, migration, inhibits apoptosis	MAPK signaling	[23]
BLACAT1	↑	Promotes proliferation, radioresistance	PSEN1	[24]
CASC-15	↑	Promotes proliferation, invasion, regulate cell cycle	miR-124, miR-33a-5p	[25,26,27]
CASC2	↑	Inhibits proliferation and tumor recurrence	miR-21	[28]
CASC-9	↑	Promotes proliferation, invasion inhibits apoptosis and autophagy	AKT/mTOR pathway, miR-423-5p/SOX12	[29,30]
CCAT-1	↑	Promotes proliferation, invasion, migration, inhibits apoptosis, regulate cell cycle	miR-181a/Wnt/β-catenin DDR2/ERK/AKT, miR155-5p, let7b-5p	[31,32,33]
CCAT-2	↑	Promotes proliferation, invasion, inhibits apoptosis	Wnt/β-catenin, GSK-3β	[34]
CEBPA-AS1	↑	Promotes tumorigenesis	CEBPA/Bcl2	[35]
CILA1	↑	Promotes EMT, drug resistance	Wnt/β-catenin signaling	[36]
DLEU1	↑	Promotes proliferation, invasion, migration and inhabits apoptosis	miR-149-5p/CDK6 HA-CD44 signaling	[37,38]
DNM3OS	↑	Promotes proliferation, migration, invasion	miR-204-5p/HIP1	[14]
ELF3-AS1	↑	Promotes proliferation	Glucose metabolism	[39]
FALEC	↓	Inhibit proliferation and migration	ECM1/EZH2	[40]
FER1L4	↑	Enhances growth, migration, invasion	miR-133a-5p/Prx1	[41]
FLJ22447	↑	CAFs activation	IL-33	[42]
FOXCUT	↑	Promotes proliferation, migration, and angiogenesis	FOXC1	[43]
FOXD2-AS1	↑	Promotes cell proliferation, migration, immunity inhibition	E2F/G2/M checkpoint	[44]
FTH1P3	↑	Enhances growth	mi-224-5p/fizzled 5	[45]
GAS5	↓	Inhibit proliferation, migration, invasion, EMT Promotes radioresistance	miR21/PTEN/PI3K/Akt	[46]
H19	↑	Promotes proliferation, invasion	miR-138/EZH2/β-catenin/GSK-3β, H19/miR-675-5p/PFKFB3	[47,48]
HAS2-AS1	↑	Induces EMT, invasion	HF-1α, NF-κB signaling	[49]
HCP5	↑	Promotes proliferation, invasion, EMT, regulate cell cycle	miR-140-5p/SOX4	[50]
HOTAIR	↑	Promotes proliferation, invasion, EMT, drug resistance, inhibits apoptosis	EZH2/H3K27me3	[9,51]
HOTTIP	↑	Promotes proliferation, invasion, migration	miR-124-3p/HMGA2/Wnt/β-Catenin	[52]
HOXA11-AS	↑	Enhances growth, proliferation, drug resistance, inhibits apoptosis	miR-214-3p, PIM1	[53]
LNC-SOX5	↑	Enhances growth, invasion, migration, inhibits apoptosis	HuR	[54]
JPX	↑	Promotes proliferation, invasion, migration	miR-944/CDH2	[55]
KCNQ1OT1	↑	Enhances proliferation, drug resistance	miR-211-5p, Ezrin/Fak/Src miR-124-3p/TRIM14	[56,57]
LBX1-AS1	↑	Inhibit proliferation and invasion	miR-182-5p/FOXO3	[58]
LEF1-AS1	↑	Promotes proliferation, invasion, inhibits apoptosis, regulates cell cycle	LATS1/YAP1, Hippo signaling	[59]
LHFPL3-AS1	↑	Promotes proliferation, invasion, migration, chemo-resistance	miR-362-5p/CHSY1	[60]
LINC00152	↑	Enhances growth, proliferation, invasion, migration	miR-139-5p	[8]
LINC00284	↑	Promotes proliferation, invasion	miR-211-3p/MAFG, FUS/KAZN	[61]
LINC00319	↑	Promotes proliferation, angiogenesis	CCL18, miR-199-5P/FZD4	[62]
LINC00460	↑	Promotes proliferation, invasion, migration, EMT	miR-320b/IGF2BP3	[63,64]
LINC00473	↑	Inhibit apoptosis	Wnt/β-catenin signaling	[65]
LINC00511	↑	Promotes proliferation, invasion	miR-765/LAMC2	[66]
LINC00668	↑	Enhances growth, proliferation	miR-297/VEGFA	[67]
LINC00673	↑	Promotes metastasis	unclear	[68]
LINC00941	↑	Promotes proliferation and tumor formation	CAPRIN2/Wnt/β-catenin signaling	[69]
LINC00958	↑	Enhances growth, proliferation, regulate cell cycle	miR-211-5p/CENPK, JAK/STAT3 signaling	[70]
LINC01133	↓	Inhibited metastasis	GDF15	[71]
LINC01137	↑	Promotes proliferation, invasion, migration	miR-22-3p	[72]
LINC01234	↑	Enhances proliferation, invasion, Inhibits apoptosis	miR-637/NUPR, miR-433/PAK4	[73,74]
LINC02195	↑	Immune regulation	MHC I	[75]
LINC-ROR	↑	Promotes proliferation	miR-145-5p, c-Myc, Klf4, Oct4, Sox2	[15]
LTSCCAT	↑	Promotes EMT, migration	miR-103a-2-5p/SMYD3/Twist1	[76]
MALAT1	↑	Promotes proliferation, invasion, EMT	SPRR, miR-125b/STAT3 NF-κB	[77,78,79]
MEG3	↓	Inhibits proliferation, invasion, migration, promotes apoptosis	Wnt/β-catenin signaling miR-548d-3p/JAK–STAT	[80,81]
MIR31HG	↑	Promotes proliferation, invasion, migration and metabolic regulation	HIF1α/p300, MMP1, BMP2, LBH	[82,83]
NKILA	↓	inhibit EMT, invasion, migration	NF-κB/Twist signaling	[84]
OIP5-AS1	↑	Enhances growth, proliferation, migration, invasion	miR-338-3p/NRP1	[85]
p23154	↑	Promote metastasis	miR-378a-3p/ GLUT1	[86]
PCAT-1	↑	Enhances growth, proliferation, invasion, migration, inhibits apoptosis	p21, c-MycAKT1-p38 MAPK signaling	[87,88]
PLAC2	↑	Promotes proliferation, invasion	H3K27 acetylation, Wnt/β-catenin signaling	[89]
PVT-1	↑	Promotes proliferation, invasion, migration, drug resistance, inhabits apoptosis	miR-150-5p/GLUT-1 miR-194-5p/HIF1a	[90,91]
RC3H2	↑	Promotes proliferation and invasion	miR-101-3p/EZH2	[92]
SLC16A1-AS1	↑	Promotes proliferation regulate cell cycle	Cyclin D1	[93]
SNHG16	↑	Promotes proliferation, invasion, inhibits apoptosis	c-Myc, miR-17-5p/CCND1	[94,95]
SNHG3	↑	Enhances proliferation, migration	miR-2682-5p/HOXB8	[96]
TIRY	↑	Induces CAFs EMT, promotes invasion and migration	miR-14/Wnt/β-catenin signaling	[97]
TUC338	↑	Enhances proliferation, regulate cell cycle, inhabits apoptosis	Unclear	[98]
TUG1	↑	Promotes proliferation, invasion, inhibits apoptosis	Wnt/β-catenin signaling	[99]
UCA1	↑	Enhances growth, proliferation, invasion, migration drug resistance, inhibits apoptosis	P27, Wnt/β-catenin, miR-184/miR-184/SF1	[100,101,102]
